# Novel methods for secondary structure determination using low wavelength (VUV) circular dichroism spectroscopic data

**DOI:** 10.1186/1471-2105-7-507

**Published:** 2006-11-17

**Authors:** Jonathan G Lees, Andrew J Miles, Robert W Janes, B A Wallace

**Affiliations:** 1 School of Biological and Chemical Sciences, Queen Mary, University of London, London E1 4NS, UK; 2 Department of Crystallography, Birkbeck College, University of London, London WC1E 7HX, UK

## Abstract

**Background:**

Circular Dichroism (CD) spectroscopy is a widely used method for studying protein structures in solution. Modern synchrotron radiation CD (SRCD) instruments have considerably higher photon fluxes than do conventional lab-based CD instruments, and hence have the ability to routinely measure CD data to much lower wavelengths. Recently a new reference dataset of SRCD spectra of proteins of known structure, designed to cover secondary structure and fold space, has been produced which includes low wavelength (vacuum ultraviolet – VUV) data. However, the existing algorithms used to calculate protein secondary structures from CD data have not been designed to take optimal advantage of the additional information in these low wavelength data.

**Results:**

In this study, we have optimised secondary structure calculation methods based on the low wavelength CD data by examining existing algorithms and secondary structure assignment schemes, and then developing new methods which have produced clear improvements in prediction accuracy, especially for beta-sheet components. We have further shown that if precise measurements of protein concentrations, and therefore spectral magnitudes, are not available, the inclusion of the low wavelength data will significantly improve the analyses. However, we have also demonstrated that the new reference dataset, methods, and assignments can also improve the analyses of conventional circular dichroism data, even if the low wavelength data is not available.

**Conclusion:**

VUV CD data include important information on protein structure which can be exploited with the algorithms and methodologies described.

## Background

Circular dichroism (CD) spectroscopy measures the differential absorbance of left- and right-handed circularly polarised light as it passes through a sample of chiral molecules. In the far ultraviolet (UV) region of the electromagnetic spectrum, the electronic transitions of amide backbone groups dominate the CD spectra of proteins, with different types of secondary structures producing characteristic spectra. Hence, the far UV CD data have been used for empirical determinations of protein secondary structure contents by employing the different reference dataset/algorithm combinations currently available [1,2]. A reference dataset consists of the CD spectra of a group of proteins, along with their corresponding secondary structure assignments derived from crystal structures. There are many methods of assigning protein secondary structures from crystallographic data including those based on C_α _coordinates [3] or hydrogen bonding patterns only [4], or in combination with phi and psi angles [5]. In addition, the Xtlsstr algorithm [6] (based on various dihedral angles) was developed with the aim of being more relevant to spectroscopic measurements. Currently, however, there is no consensus as to which of these secondary structure assignment methods correlates best with CD spectroscopic data.

The accuracy of an empirical analysis depends on the reference dataset containing representations of the types of structures present in the unknown protein [7]. Whilst existing methods tend to produce excellent results for the helical content, they are generally not very accurate in defining β-sheet and β-turn structures, and for the most part do not break down the secondary structural types into several of the components that are now seen to be functionally important, namely polyproline II (PP-II) helixes, 3_10 _helices and different types of turns.

Synchrotron radiation circular dichroism (SRCD) beamlines, which provide very bright light sources, can routinely enable the measurement of CD data to much lower wavelengths than can be achieved in conventional lab-based CD instruments [8]. Recently a new larger and broader-based reference dataset containing the SRCD spectra of proteins of known structure has been produced [9]. This contains the spectra of more than 70 proteins and has been designed for extensive coverage of both secondary structure and fold space. The components were chosen based on the CATH classification of protein structures to include representatives of all major CATH architectures and examples from each identified "superfamily" as well as to encompass the range of secondary structures present in all proteins found in the Protein Data Bank. It also incorporates low wavelength (vacuum ultraviolet – VUV) data [9]. Electronic transitions in this wavelength range also includes information on protein secondary structure [10,11], however, the existing algorithms used to calculate protein secondary structures from far UV CD data have not been designed to take optimal advantage of the additional information in the low wavelength data.

The availability of this new, significantly larger reference dataset has now provided the means by which to assess both existing and new algorithms as well as different secondary structure assignment schemes, and to examine the utility of the lower wavelength data for improving these analyses.

## Results and discussion

### Accuracy prediction indicators

The performances of CD structure determination methods are typically measured using the widely reported Pearsons correlation coefficient (*r*), and the root mean squared deviation (*δ*) (eqn.1).

δ=∑i=1n(fiCD−fiX)2n     (eqn. 1)
 MathType@MTEF@5@5@+=feaafiart1ev1aaatCvAUfKttLearuWrP9MDH5MBPbIqV92AaeXatLxBI9gBaebbnrfifHhDYfgasaacH8akY=wiFfYdH8Gipec8Eeeu0xXdbba9frFj0=OqFfea0dXdd9vqai=hGuQ8kuc9pgc9s8qqaq=dirpe0xb9q8qiLsFr0=vr0=vr0dc8meaabaqaciaacaGaaeqabaqabeGadaaakeaaiiGacqWF0oazcqGH9aqpdaGcaaqaamaalaaabaWaaabCaeaacqGGOaakcqWGMbGzdaqhaaWcbaGaemyAaKgabaGaem4qamKaemiraqeaaOGaeyOeI0IaemOzay2aa0baaSqaaiabdMgaPbqaaiabdIfaybaakiabcMcaPmaaCaaaleqabaGaeGOmaidaaaqaaiabbMgaPjabg2da9iabigdaXaqaaiabb6gaUbqdcqGHris5aaGcbaGaemOBa4gaaaWcbeaakiaaxMaacaWLjaWaaeWaaeaacqqGLbqzcqqGXbqCcqqGUbGBcqGGUaGlcqqGGaaicqaIXaqmaiaawIcacaGLPaaaaaa@4E55@

where f^CD ^= fraction of secondary structure determined from CD data, f^X ^= fraction of secondary structure calculated from the Protein Data Bank [13] (PDB) structure, and n = number of CD spectra.

In addition to these values, it is useful to consider *δ *in relation to the population standard deviation of the experimentally determined secondary structure fractions of the reference dataset (*σ*_*X*_) [12].

The ratio of *δ *to *σ*_*X *_(*ζ*) gives an indication of how much better a prediction method is than random (eqn. 2). Values of *ζ *less than 1.0 indicate that the secondary structure prediction is worse than what would be obtained from random guesses. The *ζ *parameter thus flags instances where secondary structure content analyses are meaningless.

ζ=δσX     (eqn. 2)
 MathType@MTEF@5@5@+=feaafiart1ev1aaatCvAUfKttLearuWrP9MDH5MBPbIqV92AaeXatLxBI9gBaebbnrfifHhDYfgasaacH8akY=wiFfYdH8Gipec8Eeeu0xXdbba9frFj0=OqFfea0dXdd9vqai=hGuQ8kuc9pgc9s8qqaq=dirpe0xb9q8qiLsFr0=vr0=vr0dc8meaabaqaciaacaGaaeqabaqabeGadaaakeaaiiGacqWF2oGEcqGH9aqpdaWcaaqaaiab=r7aKbqaaiab=n8aZnaaBaaaleaacqWGybawaeqaaaaakiaaxMaacaWLjaWaaeWaaeaacqqGLbqzcqqGXbqCcqqGUbGBcqGGUaGlcqqGGaaicqaIYaGmaiaawIcacaGLPaaaaaa@3DDA@

It is essential that any assessment of a dataset's predictive ability is carried out using a full cross-validation procedure. In this method the predictive performance is determined by sequentially removing a spectrum from the dataset and running the prediction method on that spectrum using the remaining spectra.

### Comparisons of algorithms

The accuracy prediction parameters were used to test and compare a number of algorithms, including several versions of SELMAT [1], one of the currently available best methods for CD analyses, several popular chemometric methods, including partial least squares (PLS), simultaneous partial least squares (SIMPLS) and principal component regression (PCR), as well as neural network (NN) and support vector machine (SVM) techniques (Tables 1, 2, 3, 4, 5). The best results (lowest *δ *or highest r) produced by any of the algorithms for each secondary structure type are shown in bold in Tables 1, 3, 4, and 5.

**Table 1:** The cross-validation performance of various algorithms using the SP175 reference dataset [9] with the standard [1] secondary structure assignment scheme.

**Dataset**	**Structure**	**SELMAT3**		**SELMAT1_norm**		**PLS**		**PLS-opt**	
		
		***δ***	***r***	***δ***	***r***	***δ***	***r***	***δ***	***r***
**SP175**	**α_R_**	0.048	0.956	0.046	0.960	**0.040**	**0.971**	0.041	0.970
	**α_D_**	**0.035**	0.809	**0.035**	**0.811**	0.036	0.791	0.037	0.779
	**β_R_**	0.073	0.792	0.064	0.849	0.063	0.853	**0.059**	**0.870**
	**β_D_**	0.020	0.913	**0.019**	**0.921**	0.023	0.889	0.025	0.867
	**turn**	0.052	0.325	0.053	0.297	0.052	**0.332**	**0.051**	0.319
	**other**	0.050	0.717	0.046	0.770	0.050	0.720	**0.045**	**0.771**
									
**SP175 (nr)**	**α_R_**	0.049	0.954	0.048	0.956	**0.041**	**0.970**	0.042	0.969
	**α_D_**	0.037	0.776	**0.036**	**0.790**	0.037	0.778	0.038	0.764
	**β_R_**	0.083	0.725	0.067	0.832	0.065	0.841	**0.061**	**0.862**
	**β_D_**	0.023	0.891	**0.021**	**0.902**	0.024	0.880	0.026	0.857
	**turn**	0.055	0.261	0.054	0.277	**0.053**	**0.302**	0.052	0.295
	**other**	0.055	0.671	0.047	0.754	0.054	0.683	**0.046**	**0.764**

The (nr) tag indicates that the cross-validation was carried out under more stringent (non-redundant) conditions such that no proteins in the training set with the same CATH homologous superfamily as that of the test protein were included. The best results (lowest *δ *or highest r) for each secondary structure type with the SP175 and SP175(nr) datasets are shown in bold.

**Table 2:** The cross-validation performances for different types of secondary structure assignments using the SP175 dataset with the PLS algorithm.

**Structure**	***δ***	***r***	***ζ***	***k***	***n***
**3_10_-helix (G)**	**0.031**	**0.385**	**1.04**	**7**	**0.04**
**β_R_**	**0.060**	**0.867**	**2.00**	**6**	**0.16**
**core β-sheet**	**0.042**	**0.879**	**2.09**	**6**	**0.13**
β-sheet _(parallel)_	0.060	0.233	0.99	6	0.02
**β-sheet _(a-parallel)_**	**0.098**	**0.806**	**1.68**	**6**	**0.07**
**β-Turn I**	**0.065**	**0.463**	**1.10**	**4**	**0.13**
β-Turn II	0.032	0.125	1.00	1	0.03
**PP-II helix**	**0.034**	**0.641**	**1.30**	**4**	**0.09**

The results shown are for the optimal number of principal components *k*·*n *is the proportion of residues in the reference dataset identified as having this type of secondary structure. Those secondary structures with ζ values greater than 1.0 are shown in bold.

**Table 3:** The cross-validation performances of various algorithms using the alternative secondary structure assignment scheme.

		**SP175**					**SP175(nr)**				
		
**Method**	**Parameter**	**α-helix**	**β_D_**	**Core β-sheet**	**PP-II helix**	**other**	**α-helix**	**β_D_**	**Core β-sheet**	**PP-II helix**	**other**
**SIMPLS**	***r***	0.968	0.895	0.875	0.687	**0.842**	0.968	0.883	0.861	0.678	**0.839**
	***δ***	0.054	0.022	0.034	0.036	**0.052**	0.055	0.023	0.036	0.036	**0.052**
**PCR**	***r***	0.966	0.894	**0.876**	0.684	0.841	0.965	0.881	**0.862**	0.677	0.837
	***δ***	0.056	0.022	**0.034**	0.036	0.052	0.057	0.023	**0.035**	0.036	0.053
**PLS**	***r***	**0.971**	0.889	0.863	0.641	0.839	**0.970**	0.881	0.854	0.628	0.833
	***δ***	**0.052**	0.023	0.035	0.038	0.052	**0.053**	0.024	0.036	0.039	0.053
**PLS-opt**	***r***	0.971	0.868	0.867	**0.702**	0.835	0.969	0.856	0.846	**0.696**	0.830
	***δ***	0.053	0.025	0.035	**0.035**	0.053	0.054	0.026	0.037	**0.035**	0.054
**SELMAT3**	***r***	0.957	0.911	0.811	0.640	0.827	0.954	0.888	0.751	0.530	0.772
	***δ***	0.063	0.021	0.041	0.039	0.054	0.065	0.023	0.047	0.043	0.062
**SELMAT1_norm**	***r***	0.958	**0.923**	0.815	0.669	0.796	0.955	**0.903**	0.774	0.668	0.771
	***δ***	0.062	**0.019**	0.040	0.037	0.058	0.065	**0.021**	0.045	0.037	0.062

The (nr) tag indicates that the cross-validation was carried out under the more stringent conditions where no proteins in the training set with the same CATH homologous superfamily as that of the test protein were included. The best results for each secondary structure type for the standard and non-redundant datasets are shown in bold.

**Table 4:** The cross-validation performances of various algorithms using the three-state α-helix (H), β-sheet (E) and other (O) assignment scheme.

**Method**	**SP175**						**SP175(nr)**					
	
	**H**		**E**		**O**		**H**		**E**		**O**	
	
	***δ***	***r***	***δ***	***r***	***δ***	***r***	***δ***	***r***	***δ***	***r***	***δ***	***r***
**SELMAT3**	0.063	0.957	0.083	0.862	0.078	0.701	0.065	0.954	0.090	0.833	0.083	0.672
**SELMAT1_norm**	0.062	0.958	0.070	0.904	0.071	0.757	0.065	0.955	0.072	0.897	0.073	0.746
**SIMPLS**	0.055	0.968	0.070	0.905	0.065	0.800	0.056	0.967	0.071	0.901	0.065	0.797
**PCR**	0.057	0.966	0.069	0.906	0.066	0.796	0.058	0.965	0.071	0.902	0.066	0.792
**PLS**	0.053	0.971	0.073	0.895	0.068	0.781	**0.053**	**0.970**	0.074	0.893	0.069	0.774
**PLS-opt**	**0.052**	**0.971**	0.070	0.902	0.066	0.796	0.054	0.970	0.072	0.900	0.066	0.790
**NN**	0.055	0.968	0.067	0.912	0.062	0.816	0.056	0.967	0.068	0.909	0.064	0.805
**SIMPL-NN**	0.057	0.965	**0.064**	**0.923**	**0.055**	**0.860**	0.056	0.964	**0.065**	**0.918**	**0.057**	**0.850**
**SVM**	0.057	0.966	0.069	0.908	0.066	0.792	0.060	0.964	0.072	0.902	0.067	0.785

The (nr) tag indicates that the cross-validation was carried out under more stringent conditions where no proteins in the training or validation set with the same CATH homologous superfamily as that of the test protein were included. The best results for each secondary structure type are shown in bold.

**Table 5:** The cross-validation performance of the NN method using various numbers of hidden neurons.

**Hidden Neurons**	**H**		**E**		**O**	
	
	***δ***	***r***	***δ***	***r***	***δ***	***r***
**1**	0.058	0.965	0.081	0.873	0.086	0.609
**3**	**0.055**	**0.968**	**0.067**	**0.912**	0.063	**0.816**
**5**	**0.055**	**0.968**	0.068	0.909	0.063	0.815
**7**	**0.055**	**0.968**	**0.067**	**0.912**	**0.062**	**0.816**
**9**	**0.055**	0.967	**0.067**	**0.912**	0.063	0.815

The secondary structure assignment scheme is the three-state α-helix (H), β-sheet (E) and other (O). The best results for each secondary structural type are highlighted in bold; they indicate that 7 neurons are marginally optimal overall for the SP175 dataset.

In the first instance, the most commonly cited secondary structure assignment method, the regular/distorted helix/sheet structure classification [14] was used to assess the methods. The new SP175 dataset has been shown to give a good prediction accuracy (low *δ*, high *r*) using this secondary structure assignment method [9] with the SELMAT3 algorithm. Improvements relative to SELMAT3 were seen for the α_R_, β_R_, turn, and 'other' fractions using the PLS or PLS-opt algorithms (Table 1). Furthermore, normalising the spectral data at each wavelength such that *μ *= 0 and *σ *= 1 before running SELMAT1 (ie. SELMAT1_norm) resulted in improvements in most of the performance accuracies relative to SELMAT3. SELMAT3 could not be used with the normalised data because the Hennessey & Johnson solution [15] for the data scaled in this way was very poor. However, SELMAT3 and SELMAT1_norm both gave greater accuracy for the α_D _and β_D _fractions relative to either of the PLS-based algorithms. But it should be noted that the α_D _and β_D _types were originally defined for use with the SELMAT3-type method.

The SIMPLS and PCR algorithms could not be tested with the standard assignment scheme because six dependent variables exceeded the maximum that could be used with these algorithms with the SP175 data.

Cross-validations were also carried out under more stringent (non-redundant) conditions such that no proteins in the training set from the same CATH homologous superfamily as that of the test protein were included (Tables 1, 3, and 4). These analyses showed little difference from those done with the dataset containing proteins from the same superfamily.

To summarise the results for the standard secondary structure assignment scheme, either of the PLS methods or SELMAT1_norm work well and generally produced better results than the SELMAT3 algorithm.

### Alternative secondary structure assignments

An important issue with respect to the assignment scheme described above is that it is not easily reconciled with the definitions of secondary structures found in the crystallographically-derived assignments produced by the DSSP algorithm (where H is an α-helix, B is an isolated β-bridge, E is a β-strand, G is a 3_10_-helix, I is a π-helix, T is a hydrogen-bonded turn, S is a bend, and O is any other type of structure) or those used by graphical packages for the display of protein structures, nor with the definitions used in sequence-based methods for prediction of secondary structures. Hence correlation of CD data with an assignment scheme that more closely relates to these definitions could find significant utility in structural biology studies. Another issue to be considered is which additional types of secondary structures could be quantitatively predicted from the SP175 dataset that have not been separately assigned by the existing scheme. As a result of both of these issues, in this study several other secondary structure assignment schemes were examined (Table 2). Analyses were done using the PLS algorithm and choosing the best cross-validated result after varying the number of principal components (*k*) from 1 to 8. The results show that β-turns II and parallel β-sheet fractions have ζ values less than or equal to 1.0, and even 3_10 _helices have values near 1.0, indicating that the predictions for these types of structures are little better than random. It is expected that the reason for this poor performance may be the small number of residues present in these conformations in the SP175 dataset (Table 2) [they are represented by only 3, 2, and 4% of the residues, respectively]. The parallel β-sheet assignment has a much poorer performance than the case where the β-sheet assignment is not sub-divided, although the separate anti-parallel sheet assignment is reasonable and provided additional information content. Filtering the DSSP β-sheet to only include residues in the core β-sheet region of the Ramachandran plot [16] produced a slightly better result than did the β_R _definition. The PP-II helical content, which has been shown to have important biological functions in a number of proteins, is reasonably well predicted.

In view of the above results a possible novel overall secondary structure assignment scheme of α-helix, β_D_, core β-sheet, PP-II helix and other was tested. The results showed high secondary structure prediction accuracies (Table 3). Four of the five types of secondary structures have r > 0.8, with even PP-II helix having a reasonable r value of ~0.7. Using this scheme, the PLS, PCR and SIMPLS methods perform similarly well and outperform the SELMAT3 method for all secondary structural types except the β_D _structures.

A simpler three-state secondary structure assignment scheme α-helix (H), β-sheet (E) and 'other' (O) (which includes G,I,T,B, and S), that has previously been shown to give good results for CD as well as FTIR data [12] was also tested. Cross-validation of the SP175 reference datasets with this secondary structure assignment show very high prediction accuracies (Table 4), with all of the 8 new methods giving better results for all structural types compared to SELMAT3.

### Neural network (NN) and support vector machine (SVM) methods

For neural network methods, the number of network weights must be kept to a minimum. Hence only the three-state assignment scheme was used. The performance of the neural network trials undertaken with varying numbers of hidden layer nodes greater than 1 gave similar results (Table 5), with 7 hidden nodes giving marginally the best overall performance. Using the extra inputs to the neural network from the SIMPLS helix and sheet predictions (SIMPL-NN) improved their prediction accuracies further (Table 4). The best NN performance was for the SIMPL-NN algorithms and, indeed, this produced the best overall results for the three-state model.

### Effect of low-wavelength cut-off

**Figure 1:**
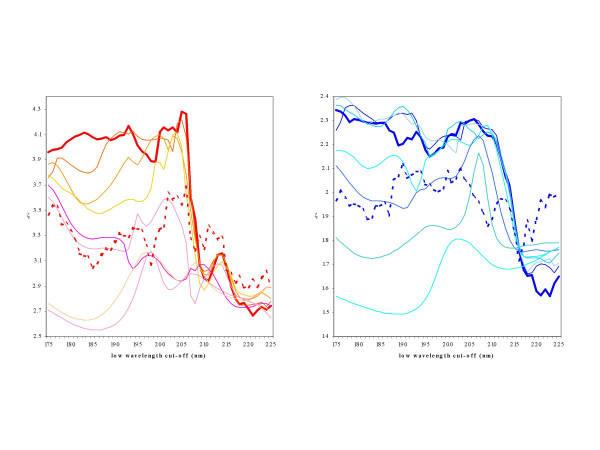
**The effect of low-wavelength cut-off on performance accuracy**. The ζ parameter was calculated for the SIMPLS algorithm using various low wavelength cut-offs applied to the SP175 dataset. In shades of red are the performance curves for α-helices (H) and in shades of blue are those for β-sheets (E). The thick solid lines indicate the performance using *k *= 8 (ie. 8 principal components). The thin lines are derived using progressively smaller values of *k*. The dashed lines are for values calculated using SELMAT3 instead of SIMPLS.

The effect of the low wavelength cut-off on the SIMPLS, PLS, PCR and SELMAT3 algorithms was assessed. SIMPLS, PLS and PCR algorithms gave similar results so only results from SIMPLS are shown (Figure 1). If 8 principal components are used, the results for helical secondary structures are relatively insensitive to the low-wavelength cut-off as long as data to 205 nm is included. When the low-wavelength cut-off is above ~205 nm there is a massive drop in the prediction accuracies. For β-sheet determinations, there is a slow but significant decrease in performance over the region from 175 to 208 nm, suggesting the availability of the low wavelength data may be more important for accurate analyses of β-sheets, a type of secondary structure not particularly well-analyzed with only far UV data. None of the alternative values of *k *for the algorithms are able to prevent the drop in performance in this region so the effect is not due to including either an excessive or inadequate number of principal components. Hence it would appear that for general use of the SIMPLS algorithm, k = 8 is the optimal value for the SP175 dataset. For 3_10 _helices, there is little difference in the accuracy with the inclusion of the low wavelength data (data not shown), however, for PP-II helices, the accuracy is, like β-sheets, slightly improved as more low wavelength data is included.

**Figure 2:**
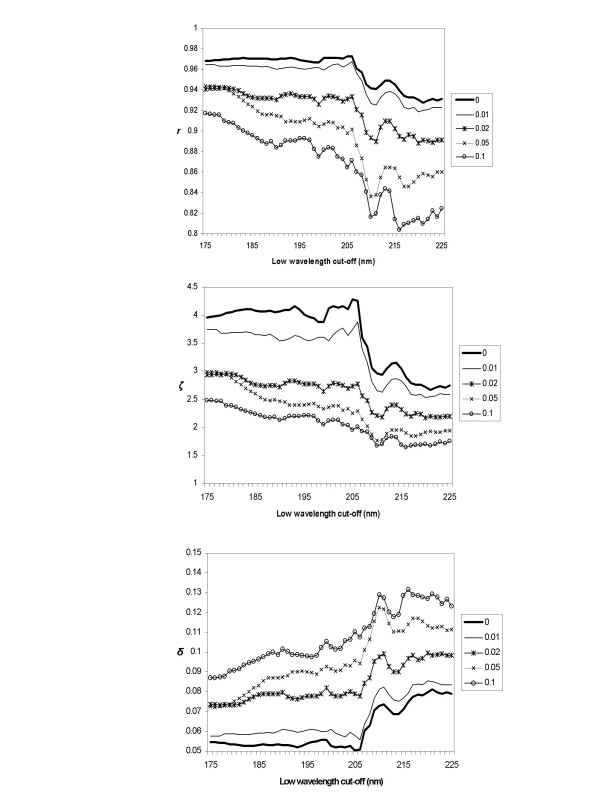
**a-c – The effect of low wavelength cut-off as a function of increasing errors in magnitude**. An example of the effect of the low-wavelength cut-off as a function of increasing errors in spectral magnitude on the performance as judged by the parameters A) ζ, B) r, and C) δ, respectively, using the SIMPLS algorithm with the SP175 dataset. The different curves on each plot represent different results obtained after applying progressively larger scale factors to represent errors in magnitude. The numbers represent the variance of the normal distribution from which the scaling factors were randomly chosen. These were for the α-helix (H) secondary structure component as assigned by DSSP.

These modest wavelength cut-off results are somewhat surprising given that the peptide backbone produces a number of electronic transitions below 205 nm. This suggests that perhaps the magnitudes of the peaks at wavelengths higher than 205 nm dominate the analyses. To test this, the spectral shapes were left unchanged but scale factors of different magnitudes were applied to change the spectral magnitudes and the analyses were repeated. This was done because an important practical consideration in analyses of CD spectra is the correctness of the spectral magnitude. Significant errors in magnitude can arise from inaccurate determinations of protein concentration or optical cell pathlength [17,18]. To understand the effects of these errors on the accuracy, analyses as a function of low wavelength cut-off were undertaken. The results (Figure 2) show that for the α-helix secondary structure the cross-validated performance of the correctly scaled SP175 dataset was similar to that of the SP175 dataset with a small scaling error added (variance = 0.01) for all the low wavelength cutoff values used. However, as progressively greater magnitude scale factors were applied, the cross-validated performance accuracy became more dependent on the low-wavelength cut-off value. For instance, when errors on the order of only 10% are present, the improvement in accuracy with the addition of low wavelength data is quite dramatic: the value of *r *increases from 0.86 to 0.92. This suggests that the lack of correlation of low-wavelength data cut-off with cross-validated performance of the SP175 dataset can be partly attributed to the high accuracy of the concentration and pathlength determinations in the component spectra of the dataset. It also indicates that when the SP175 dataset is used for analyses of other proteins, the low wavelength data will provide some robustness against magnitude error.

## Conclusion

We have described several novel algorithms, including support vector machine and neural network methodologies, that produced higher accuracies for secondary structure determination than those currently in use for CD analyses. The SELMAT algorithms remain the best for predicting the α_D _and β_D _secondary structures, but these classifications are not easily correlated with standard secondary structure categories.

We have shown which structures can and cannot be quantitatively determined from the CD spectra of the new larger and broader-based SP175 dataset (Table 2). Excluding non-core β-sheet residues from the β-sheet fraction makes sense in view of the large variety of dihedral angles assigned as β-sheet by the DSSP algorithm. This method improves the general prediction of the remaining secondary structures. A simple 3-state prediction of α-helix (H), β-sheet (E) and 'other' (O, which includes G,I,T,B, and S) has been shown to give very good quantitative prediction for all fractions (Table 3). With this method α-helix (H) and β-sheet (E) have values of *r *as high as 0.97 and 0.92, respectively. The alternative three-state assignment scheme discussed here mimics the types of secondary structures described for crystal structures and identified by graphics programs and sequence/secondary structure prediction programs more closely than some of the more obscure assignment methods often used for CD data analyses. It is anticipated that this will be a robust method since proteins with high 3_10 _helix (G) and β-bridge (B) content will not necessarily have an α-helical or β-sheet type spectrum because of length effects. Also this secondary structure assignment method can be easily applied in conjunction with three-state sequence prediction methods using a compatible secondary structure assignment algorithm such as the SSPro8 server [19]. The alternative secondary structure assignments of α-helix (H,G,I), β-sheet (E,B) and 'other' (T,S,C) would need to be used with some commonly-used sequence-based structure prediction methods [20]. We would expect the H, E, O assignment to be better in cases where proteins have large amounts of G relative to H, or large amounts of B relative to E since length effects will be important in these instances. It should be noted, however, that the SP175 dataset is particularly lacking in proteins with significant disorder (in part because there is a dearth of good structures of such proteins in the PDB), so these types of structures have not been separated out from "other" components in this study.

In order to examine the effect of spectral redundancy in the reference dataset, the accuracies of the methods were tested with redundant and non-redundant versions of the reference dataset. In this study, there was little degradation of either the analysis quality associated with removal of these data from the reference dataset nor was there a change in the ordering of which method works best. Hence, the improvements described do not arise from the algorithms exploiting structural redundancy in the dataset. However, the results do suggest that in the future the most valuable additions to the reference dataset will be from proteins with unique structures.

All of the methods implemented in this paper, with the exception of SELMAT, are only capable of mapping linear relationships in the data. The neural network and SVR methods used linear transfer/kernel functions, respectively. As the SP175 reference dataset is expanded in the future by supplementation with additional spectra, it may be possible to use non-linear versions of the SVR (polynomial kernel) and NN (sigmoidal transfer function) which are more demanding of input data to allow mapping of non-linear relationships.

Paradoxically, an important result emerges in the sharp cut-off of prediction accuracy observed when the broadly-based dataset is truncated to a low-wavelength cut-off at ~205 nm. With SRCD it is possible to collect data to ≤ 205 nm in almost any solvent and buffer commonly used in biological studies, including 6 M guanidine hydrochloride and urea. Thus, these methods should provide especially good improvements for assessing secondary structure in protein folding and unfolding studies.

A final, and very significant result, which will find practical application in CD analyses, is that when accurate information on protein concentration is unavailable (very often the case), the inclusion of the low wavelength VUV data will produce much better analyses than if only the far UV data is used.

In conclusion, the SIMPLS and PLS methods appear to work consistently amongst the best methods with all of the secondary structure assignment schemes tested. For the three-state (H,E,O) scheme, the more complicated SIMPL-NN produced the best overall results. However, due to its much greater simplicity, the SIMPLS method should be preferred over the SIMPL-NN method until a larger CD reference dataset is available.

## Methods

### Reference dataset

The SP175 dataset currently contains SRCD spectra for 72 proteins with a low wavelength cut-off at or below 175 nm [9]. It was designed to extensively cover secondary structure and fold space, and to combine high quality spectroscopic data with high resolution, well-defined crystal structure data.

### Secondary structure assignments

The DSSP algorithm [4] was used to assign secondary structures from the PDB files [13]. The helical and sheet secondary structures were further divided into distorted and regular helices (α_D_,α_R_) and distorted and regular sheet (β_D_, β_R_) classes, as previously defined [14]. Any residues not present in the crystal structures were assigned to the 'other' fraction. A script was written to implement the PP-II helix assignment method previously employed for CD analyses [21]. For this we chose to use the less stringent criterion, allowing PP-II helix assignment even for PP-II helix stretches of 1 residue in length.

As an alternative means of division into secondary structural types, the "core" β-sheet structures were assigned to those residues designed as β-sheet (E) by DSSP and also lying in the "most favoured" β region [16] of the Ramachandran map. This area was taken to be an ellipse centred at φ = -120° φ = 135°, with major and minor axis lengths of 100° and 55°, respectively. The axis of the ellipse was parallel to the main diagonal (top left, bottom-right) of the Ramachandran map. β-turn secondary structure assignments were implemented using the definitions of PROMOTIF [22]. The parallel and antiparallel β-sheets were assigned using the assignment from the DSSP algorithm. Three of the four characteristic β-turn backbone angles were allowed to deviate by ± 30° and one by ± 40° from the ideal values. The hierarchy of secondary structure assignment for β-turn was α-helix (H) > β-sheet (E) > β-turn I > β-turn II > 'other'. n is the number of residues in the reference dataset proteins identified as having a particular type of secondary structure divided by the total number of residues (22,372) in the dataset proteins.

### Calculation algorithms

Several different methods were compared: A re-implementation of SELCON3 [14] (SELMAT3), described previously [9] was used as a representative of the currently available best methods, all of which have been shown to have a similar accuracy [23]. SELMAT1 is the stage of the SELMAT3 algorithm before application of the spectral fitting rule and so corresponds to the CDPro SELCON1 algorithm [24].

To assess the effects of data normalisation, the SP175 dataset was scaled so that the CD values at each wavelength had a zero mean (*μ*) and a standard deviation (*σ*) of 1 [12]. (In other words, each individual wavelength first had the mean subtracted, then there was a subsequent scaling so that the standard deviation of the CD measurements was 1.0) When this dataset was used with the SELMAT1 algorithm, the method was referred to as SELMAT1_norm.

Methods of analyses widely used in the field of chemometrics include partial least squares (PLS), simultaneous partial least squares (SIMPLS) and principal component regression (PCR). These have been previously tested on a limited CD dataset [12] but the algorithms are not currently available for CD analyses. In this study, these algorithms were accessed using the 'csimpls' and 'cpcr' functions implemented in the freely available LIBRA package [25] for MATLAB [26] and tested with our large and broadly-based CD dataset SP175. The default number of principal components (*k*) used in the calculations reported in this study was set to be the same as the information content calculated for the SRCD reference datasets (*k *= 8) [9].

An alternative version of the PLS algorithm, designated as PLS-opt, was developed using an extra cross-validation step to find the optimal value of *k*. After the test protein was removed from the dataset the second series of cross-validations was carried out on the remaining proteins with *k *values varied from 4 to 8. The value of *k *found to give the best result was then used with the PLS algorithm to analyze the test protein.

Backpropagation neural networks (NN) have previously been implemented for secondary structure prediction by CD [27]. However, the numbers of weights in the network were very high in comparison to the number of training patterns due to the limited number of proteins in the reference dataset. In addition, the reported prediction accuracies were on the validation set rather than the test set. Under these conditions it is very common to overfit the data. In the implementation described here, we used the new larger SP175 reference dataset and the Levenberg-Marquardt backpropagation method which is better suited to relatively small datasets. Although the dimensions of the input vectors are quite large (points at 1 nm intervals from 240–175 nm) the variables of the CD spectra are highly correlated. In this situation it is useful to reduce the dimensionality of the data using principal component analysis (PCA). This allows the number of input neurons in the neural network to be kept to a minimum. Before being subjected to PCA, the dataset was normalised as described above so that the CD values at each wavelength had *μ *= 0, *σ *= 1 (a common procedure before PCA). Only the six most significant principal components were retained since these components accounted for all of the gross features of the data and because it is important to keep the number of free parameters small for NN. The inputs to the network were then all scaled to fall in the range [+1,-1]. The hidden layer and output transfer functions were both linear, thus creating a smoother error surface, which simplifies NN training. The training was carried out using full cross-validation. At each stage of the cross-validation 10% of the training set was removed and used as the validation set. Over-training was prevented by stopping the network at the point where the validation set mean squared error of prediction started to increase. The analysis of the test protein CD spectrum by the neural network was then carried out. This testing procedure satisfies the criterion that the testing data is not used in the training or validation steps. The performance of the network was evaluated for 1, 3, 5, 7 and 9 hidden neurons. After finding the optimal number of hidden neurons, the training/validation/testing procedure was repeated with the α-helix and β-sheet predictions of the SIMPLS algorithms given as two additional inputs to the neural network (SIMPL-NN).

Support vector machines (SVMs) were created as an additional method for secondary structure prediction using the linar kernel function to carry out epsilon support vector regression implemented in the LibSVM v2.4 package [28]. The SP175 dataset was processed in the same way as for the neural networks. Assessment of the SVM performance was carried out by leave-one-out cross-validation. After the test protein was removed from the dataset, the remaining protein spectra were used to determine the optimal *C *and *rho *parameters for the SVM [28]. Repeated 7-fold cross-validations of the remaining protein spectra were calculated. At no point was the test protein used in optimising the SVM parameters for its own analysis.

The cross-validated values from the SIMPLS, PLS, PCR, NN, SIMPL-NN and SVM algorithms were adjusted so that any predicted negative fractions were set to 0%. The remaining secondary structure fractions were then rescaled to give a total of 100%, resulting in constrained, normalized solutions.

Non-redundant cross-validations [14], designated (nr), were implemented for each of the methods. These assess the effects of removing proteins with homology to the test protein from the reference dataset during the cross-validation procedure, so that an approximation of potential effects from structural redundancy in the dataset can be determined. This was accomplished by removing any protein in the training set from the same CATH homologous superfamily [29] as that of the test protein.

### Low wavelength effects as a function of concentration uncertainty

Numbers were randomly drawn from a normal distribution with a mean of 1.0 and a given variance for each of the spectra in the SP175 dataset. Each spectrum was then scaled by its corresponding random value to generate a new dataset with extra scaling error in comparison to the SP175 dataset. This was repeated several times where the variance of the normal distribution from which the random scaling factors was chosen ranged from 0.01 to 0.10. Each of the datasets generated was then cross-validated a number of times with different low wavelength cut-off values.

### Availability and requirements

#### Algorithms

Project name: Algorithms for CD Spectroscopic Analyses

Project home page: 

Operating system(s): Windows (2000 and later versions) or Linux

Programming language: Perl

Licence: Scripts are available free (no licence required) from this site as a zipfile, and include a README.txt file with instructions for use. Requirement that the user has access to MATLAB and LIBRA MATLAB.

Restrictions to non-academics: None

#### Reference data set

The SP175 reference data set is available as noted in the paper describing its creation [9], namely in the Dichroweb webserver [2] located at  and will be available in the Protein Circular Dichroism Data Bank (PCDDB) website [30] located at .

## Authors' contributions

JGL wrote the computing scripts and did most of the calculations, AJM did some of the calculations, and RWJ and BAW directly supervised this work. All authors participated in the writing and analysis, and have read and approved of the manuscript. None of the authors have any competing financial or other interests in relation to this work.
